# Supplementary material for: Treatment heterogeneity of water, sanitation, hygiene, and nutrition interventions on child growth by environmental enteric dysfunction and pathogen status for young children in Bangladesh
Source: PLoS Negl Trop Dis. 2025 Feb 18;19(2):e0012881. doi: 10.1371/journal.pntd.0012881 (PMC11882089; doi:10.1371/journal.pntd.0012881)
Supplement: S1 Fig — N+WSH, combined nutrition, water, sanitation, and hygiene intervention. (DOCX) [file pntd.0012881.s001.docx]

**S1 Fig. Correlation of individual N+WSH conditional treatment effect and myeloperoxidase concentration**

**
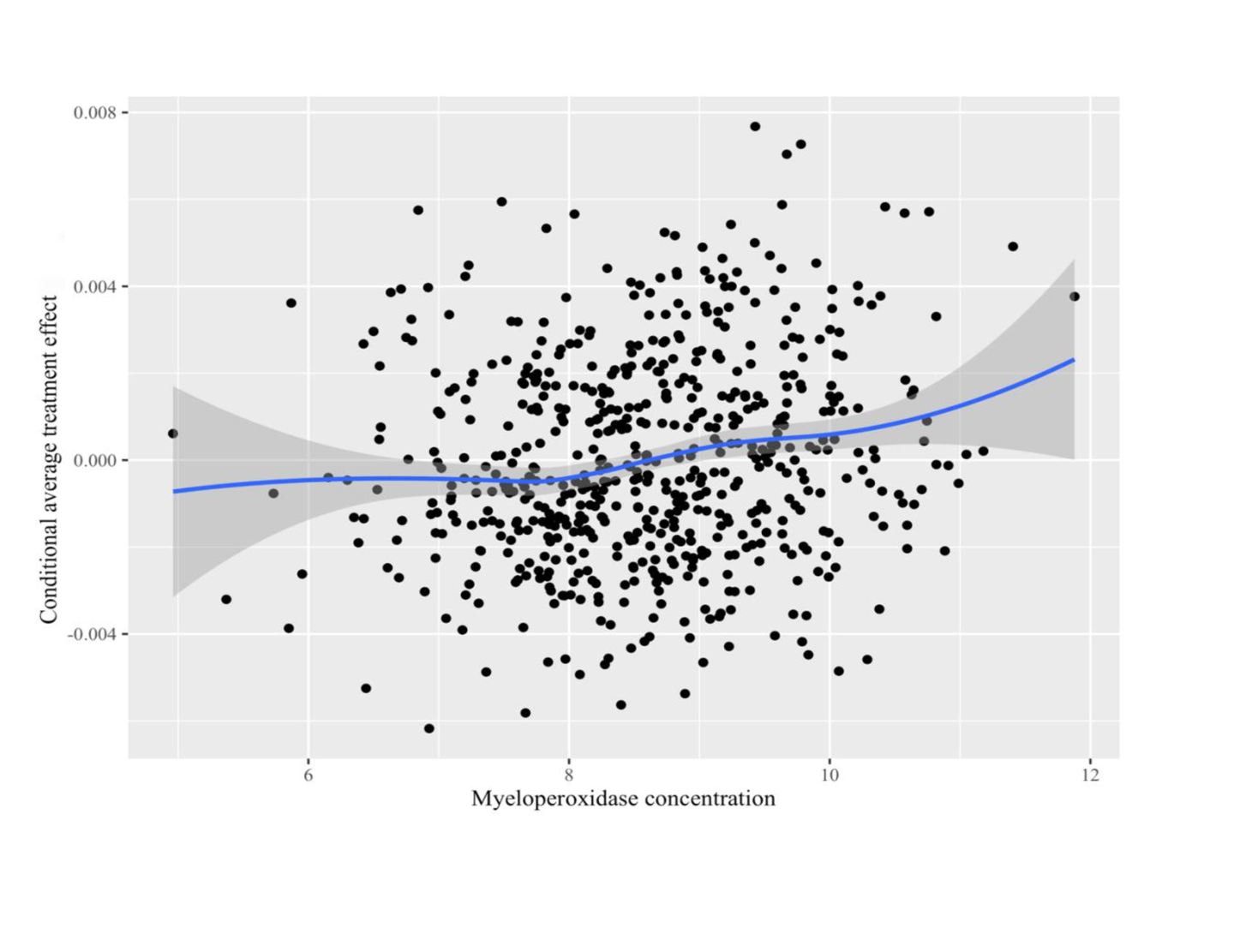
**

N+WSH, combined nutrition, water, sanitation, and hygiene intervention
