# Supplementary material for: Treatment heterogeneity of water, sanitation, hygiene, and nutrition interventions on child growth by environmental enteric dysfunction and pathogen status for young children in Bangladesh
Source: PLoS Negl Trop Dis. 2025 Feb 18;19(2):e0012881. doi: 10.1371/journal.pntd.0012881 (PMC11882089; doi:10.1371/journal.pntd.0012881)
Supplement: S1 Table — (DOCX) [file pntd.0012881.s007.docx]

**S1 Table. Biomarker and Pathogen Correlation with NWSH Conditional Average Treatment Effect, Excluding Children with Diarrhea at Year 1**

| Biomarker or pathogen | Treatment effect (HAZ difference) at non-detection (pathogen) or below median (EED biomarker) | Treatment effect (HAZ difference) at detection (pathogen) or above median (EED biomarker) | Difference in Treatment effect (95% CI) |
| --- | --- | --- | --- |
| Any enterotoxigenic *Escherichia coli* | -0.0006 | 0.0012 | 0.0018 (0.0017,0.002) |
| *Campylobacter jejuni/coli* | -0.0004 | 0.0011 | 0.0015 (0.0014,0.0017) |
| *Campylobacter* spp. | -0.0004 | 0.0006 | 0.001 (0.0008,0.0011) |
| REG 1B | -0.0004 | 0.0002 | 0.0005 (0.0004,0.0007) |
| Myeloperoxidase | -0.0004 | 0.0003 | 0.0007 (0.0005,0.0008) |
| Any enteropathogenic *Escherichia coli* | 0.0005 | -0.0004 | -0.0009 (-0.001,-0.0007) |
| Alpha-1-antitrypsin | 0.0007 | -0.0008 | -0.0015 (-0.0016,-0.0013) |
| Enteroaggregative *Escherichia coli* | 0.0012 | -0.0003 | -0.0015 (-0.0016,-0.0013) |
| Atypical enteropathogenic *Escherichia coli* | 0.0006 | -0.0013 | -0.0019 (-0.002,-0.0017) |
