# Supplementary material for: Treatment heterogeneity of water, sanitation, hygiene, and nutrition interventions on child growth by environmental enteric dysfunction and pathogen status for young children in Bangladesh
Source: PLoS Negl Trop Dis. 2025 Feb 18;19(2):e0012881. doi: 10.1371/journal.pntd.0012881 (PMC11882089; doi:10.1371/journal.pntd.0012881)
Supplement: S8 Fig — Figure created using BioRender [1]. (DOCX) [file pntd.0012881.s009.docx]

**S8 Fig. Study timeline. Created using Biorender [1].**
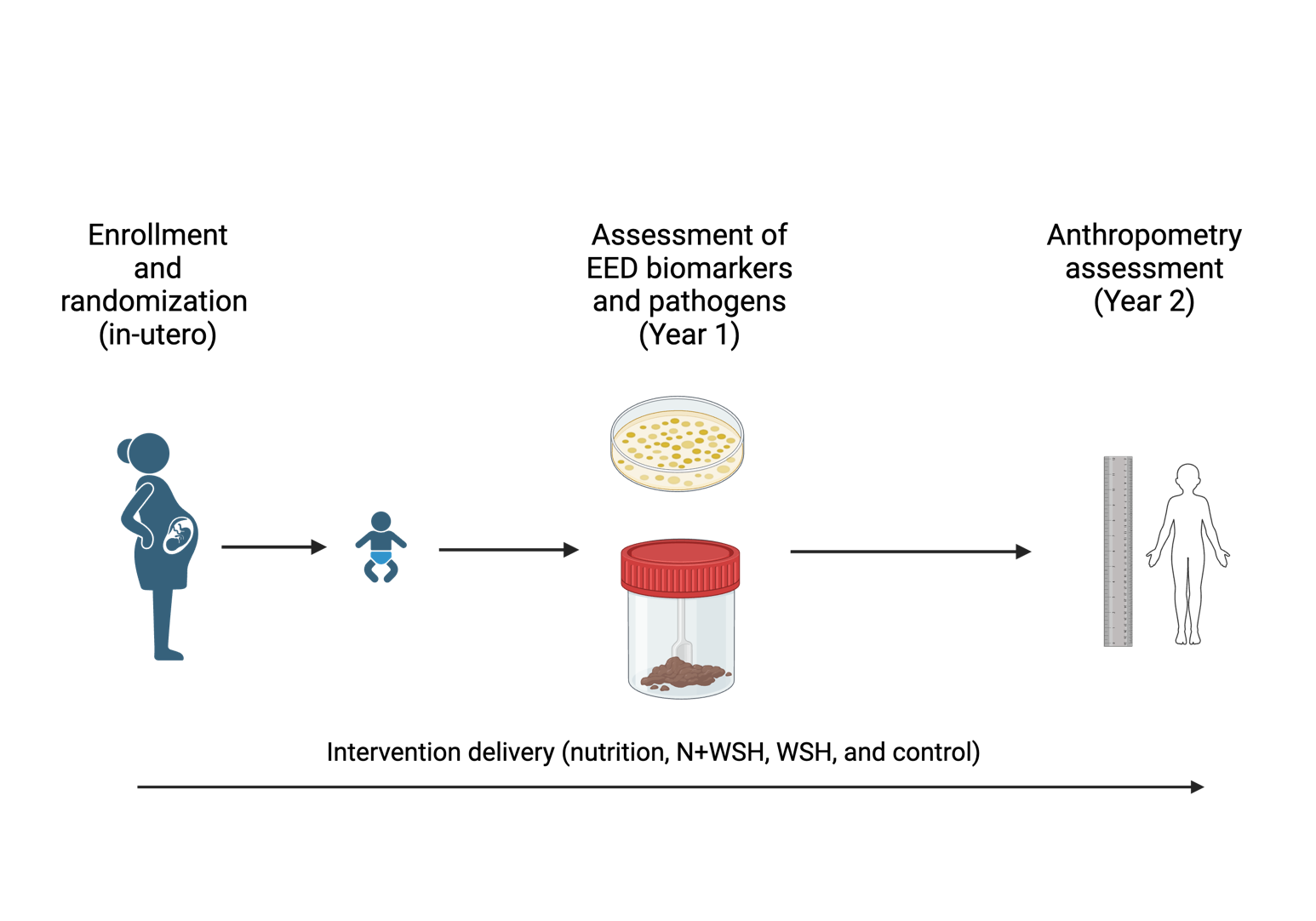


References

1. Created with Biorender.com. 2024. Available: ‍https://biorender.com
