# Supplementary material for: Treatment heterogeneity of water, sanitation, hygiene, and nutrition interventions on child growth by environmental enteric dysfunction and pathogen status for young children in Bangladesh
Source: PLoS Negl Trop Dis. 2025 Feb 18;19(2):e0012881. doi: 10.1371/journal.pntd.0012881 (PMC11882089; doi:10.1371/journal.pntd.0012881)
Supplement: S1 Text — (DOCX) [file pntd.0012881.s010.docx]

**S1 Text. Study interventions.**

We included four study arms: control, combined water treatment, sanitation, and handwashing (WSH), nutrition, and nutrition plus WSH (N+WSH). The control arm was passive, including no visit by a health promoter. The water treatment involved provision of chlorine tablets (Aquatabs; NaDCC) and a safe storage vessel to treat and store drinking water. Sanitation involved upgrading latrines to double pit latrines for all households in study compounds, providing child potties, and sani-scoops to remove feces from households and compounds. The handwashing intervention involved providing handwashing stations near latrines and kitchens, which included soapy water bottles and detergent soap. The nutrition intervention involved the daily provision of a small-quantity lipid-based nutrient supplement and age-appropriate recommendations on maternal nutrition and child feeding. All interventions were delivered until the child was 24 months of age.

Intervention promoters, who were residents of the study area, visited participants to promote intervention behaviors at the level of the compound (cluster of nearby houses). Each promoter received at least five days of training prior to visiting compounds, and received periodic refresher courses throughout the intervention period. These promoters used various strategies to promote intervention behaviors. For example, promoters promoted the hygiene intervention (handwashing) by framing it as a nurturing intervention that was facilitated by the handwashing station and soap provided by the intervention [1,2].

References

1. Arnold BF, Null C, Luby SP, Unicomb L, Stewart CP, Dewey KG, et al. Cluster-randomised controlled trials of individual and combined water, sanitation, hygiene and nutritional interventions in rural Bangladesh and Kenya: the WASH Benefits study design and rationale. BMJ Open. 2013;3: e003476–e003476. doi:10.1136/bmjopen-2013-003476

2. Curtis VA, Danquah LO, Aunger RV. Planned, motivated and habitual hygiene behaviour: an eleven country review. Health education research. 2009;24: 655–673.
