# Supplementary material for: Treatment heterogeneity of water, sanitation, hygiene, and nutrition interventions on child growth by environmental enteric dysfunction and pathogen status for young children in Bangladesh
Source: PLoS Negl Trop Dis. 2025 Feb 18;19(2):e0012881. doi: 10.1371/journal.pntd.0012881 (PMC11882089; doi:10.1371/journal.pntd.0012881)
Supplement: S2 Text — (DOCX) [file pntd.0012881.s011.docx]

**S2 Text. Inclusion criteria.**

We included households if a resident was a pregnant mother in her first or second trimester, the household was located in a rural area that was not fully submerged during monsoon season and did not have water, sanitation, hygiene, or nutrition programs ongoing or planned in the next two years.

We excluded households whose residents had plans to move during the following year, did not own their home, or drew water from a source with high iron content or high arsenic content.

We included child participants who were born to enrolled mothers meeting household inclusion criteria within six months of the baseline survey. We excluded children if their growth score fell outside of the WHO plausible range [1].

References

1. de Onis M, Onyango AW, Van den Broeck J, Chumlea WC, Martorell R. Measurement and standardization protocols for anthropometry used in the construction of a new international growth reference. Food Nutr Bull. 2004;25: S27-36. doi:10.1177/15648265040251S104
