# Supplementary material for: Treatment heterogeneity of water, sanitation, hygiene, and nutrition interventions on child growth by environmental enteric dysfunction and pathogen status for young children in Bangladesh
Source: PLoS Negl Trop Dis. 2025 Feb 18;19(2):e0012881. doi: 10.1371/journal.pntd.0012881 (PMC11882089; doi:10.1371/journal.pntd.0012881)
Supplement: S3 Text — (DOCX) [file pntd.0012881.s012.docx]

**S3 Text**. **Adjustment covariates.**

Enrollment characteristics: child sex (male or female), child birth order (first born, second born or greater), maternal age (years), maternal height (cm.) maternal education level (no education, primary, or secondary/greater), household food insecurity (4-level HFIAS categories), number of children in the household less than 18 years old, total number of individuals living in the compound (group of nearby houses), household’s distance to primary drinking water source (in minutes), household construction materials (floor, walls, and roof), and an asset-based household wealth index, calculated from the first principal component of a principal components analysis of household assets (electricity, wardrobe, table, chair or bench, khat, chouki, working radio, working black/white or color television, refrigerator, bicycle, motorcycle, sewing machine, mobile phone, land phone, number of cows, number of goats, number of chickens).

Time-varying characteristics: child age (days) at assessment and month of data collection.
