# Supplementary material for: Treatment heterogeneity of water, sanitation, hygiene, and nutrition interventions on child growth by environmental enteric dysfunction and pathogen status for young children in Bangladesh
Source: PLoS Negl Trop Dis. 2025 Feb 18;19(2):e0012881. doi: 10.1371/journal.pntd.0012881 (PMC11882089; doi:10.1371/journal.pntd.0012881)
Supplement: S4 Text — (DOCX) [file pntd.0012881.s013.docx]

**S4 Text. Laboratory methods.**

*Fecal EED Biomarkers and pathogens*

Collection and assay procedures have been described previously [1]. The child’s primary caregiver collected the fecal sample, and it was placed on cold chain at median time 155 minutes (interquartile range, [IQR], 80–529), and then transported on dry ice to the laboratory where it was stored at -80 degrees Celsius [2]. We extracted DNA and RNA using QIAamp Fast DNA Stool Mini kit (Qiagen, Venlo, The Netherlands) as well as spike-ins of two extrinsic controls which aimed to assess efficiency of extraction and amplification . We assessed enteropathogens at icddr,b using quantitative polymerase chain reaction (PCR) via TaqMan array card [3,4]. We quantified pathogens using quantification cycle, where one unit corresponded to twice the pathogen quantity and there was an analytical limit of detection at quantification cycle 35 [5]. These quantities were normalized based on the efficiency of per-sample extraction/amplification.

icddr,b researchers measured fecal alpha-1-antitrypsin, myeloperoxidase, neopterin, and REG1B following ELISA kit protocols [1]. The initial dilutions were 1:500 for myeloperoxidase (Alpco, Salem, NH), 1:75 in 0.9% saline for neopterin (GenWay Biotech, San Diego, CA), 1:25000 for alpha-1-antitrypsin (BioVendor, Asheville, NC), and 1:40000 for REG1B (TechLab, Blacksburg, VA). If specimens were out-of-range, they were rerun at alternate dilutions, and samples that were below the standard curve were rerun without dilution.

Laboratory analysts were masked, but participants, outcome assessors, and data analysts could not be masked.

References

1. Lin A, Ali S, Arnold BF, Rahman MZ, Alauddin M, Grembi J, et al. Effects of Water, Sanitation, Handwashing, and Nutritional Interventions on Environmental Enteric Dysfunction in Young Children: A Cluster-randomized, Controlled Trial in Rural Bangladesh. Clinical Infectious Diseases. 2020;70: 738–747. doi:10.1093/cid/ciz291

2. Grembi JA, Lin A, Karim MA, Islam MO, Miah R, Arnold BF, et al. Effect of water, sanitation, handwashing and nutrition interventions on enteropathogens in children 14 months old: a cluster-randomized controlled trial in rural Bangladesh. J Infect Dis. 2020. doi:10.1093/infdis/jiaa549

3. Liu J, Gratz J, Amour C, Nshama R, Walongo T, Maro A, et al. Optimization of quantitative PCR methods for enteropathogen detection. PloS one. 2016;11: e0158199.

4. Liu J, Platts-Mills JA, Juma J, Kabir F, Nkeze J, Okoi C, et al. Use of quantitative molecular diagnostic methods to identify causes of diarrhoea in children: a reanalysis of the GEMS case-control study. The Lancet. 2016;388: 1291–1301.

5. Platts-Mills JA, Liu J, Rogawski ET, Kabir F, Lertsethtakarn P, Siguas M, et al. Use of quantitative molecular diagnostic methods to assess the aetiology, burden, and clinical characteristics of diarrhoea in children in low-resource settings: a reanalysis of the MAL-ED cohort study. The Lancet Global Health. 2018;6: e1309–e1318.
