# Supplementary material for: Treatment heterogeneity of water, sanitation, hygiene, and nutrition interventions on child growth by environmental enteric dysfunction and pathogen status for young children in Bangladesh
Source: PLoS Negl Trop Dis. 2025 Feb 18;19(2):e0012881. doi: 10.1371/journal.pntd.0012881 (PMC11882089; doi:10.1371/journal.pntd.0012881)
Supplement: S2 Table — (DOCX) [file pntd.0012881.s014.docx]

**S2 Table. Pathogens, EED biomarkers, covariates, and reasons for exclusion.[6]**

| **Variable** | **Inclusion** | **Exclusion Reason** | **Gene Target** |
| --- | --- | --- | --- |
| **Pathogens** | | |  |
| *Campylobacter jejuni/coli* | Included | NA | *cadF* |
| Enteroaggregative *Escherichia coli* (EAEC) | Included | NA | *aaiC* or *aatA*, or both |
| Atypical enteropathogenic *E. coli* (aEPEC) | Included | NA | *eae* without *bfpA, stx1*, and *stx2* |
| *Campylobacter* spp. | Included | NA | *cpn60* |
| Any Enterotoxigenic *E. coli* | Included | NA | *LT, STh,* or *STp* |
| Any Enteropathogenic *E. coli* (EPEC) | Included | NA | *bfpA* and *eae* (tEPEC)  or  *eae* without *bfpA, stx1*, and *stx2* (aEPEC) |
| Typical enteropathogenic *Escherichia coli* (tEPEC) | Excluded | Near zero variance | *bfpA* and *eae* |
| Shiga toxin–producing *E. coli* | Excluded | Near zero variance | *eae* without *bfpA* and with *stx1, stx2*, or both |
| *Shigella/*enteroinvasive *E. coli* | Excluded | Near zero variance | *ipaH* |
| *Ancyclostoma duodenale* | Excluded | Near zero variance | *ITS2* |
| *Necator americanus* | Excluded | Near zero variance | *ITS2* |
| *Entocytozoon bieneusi* | Excluded | Near zero variance | *ITS* |
| *Encephalitozoon intestinalis* | Excluded | Near zero variance | SSU rRNA |
| *Entamoeba histolytica* | Excluded | Near zero variance | 18S rRNA |
| *Entamoeba* spp. | Excluded | Near zero variance | 18S rRNA |
| *Giardia* spp. | Excluded | Near zero variance | 18S rRNA |
| *Cryptosporidium* spp. | Excluded | Near zero variance | 18S rRNA |
| *Salmonella enterica* | Excluded | Near zero variance | *ttr* |
| *Hymenolepis nana* | Excluded | Near zero variance | *ITS1* |
| *Schistosoma spp.* | Excluded | Near zero variance | *ITS* |
| *Bacteroides fragilis* | Excluded | Near zero variance | EGBF |
| *Helicobacter pylori* | Excluded | Near zero variance | *ureC* |
| Rotavirus | Excluded | Near zero variance | *NSP3* |
| *Ascaris lumbricoides* | Excluded | Near zero variance | *ITS1* |
| *Trichuris trichuria* | Excluded | Near zero variance | 18S rRNA |
| *Cyclospora spp.* | Excluded | Near zero variance | 18S rRNA |
| *Isospora spp.* | Excluded | Near zero variance | 18S rRNA |
| *Cryptosporidium hominis* | Excluded | Near zero variance | 18S rRNA |
| *Cryptosporidium parvum* | Excluded | Near zero variance | 18S rRNA |
| *Strongyloides spp.* | Excluded | Near zero variance | Dispersed repetitive sequence |
| *Blastocystis spp.* | Excluded | Near zero variance | 18S rRNA |
| *Vibrio cholerae* | Excluded | Near zero variance | *hlyA* |
| *Mycobacterium tuberculosis* | Excluded | Near zero variance | *IS6110* |
| *Clostridium difficile* | Excluded | Near zero variance | *tcdA, tcdB* |
| *Plesiomonas spp.* | Excluded | Near zero variance | *gyrB* |
| *Aeromonas spp.* | Excluded | Near zero variance | Aerolysin |
| Astrovirus | Excluded | Near zero variance | Capsid |
| Norovirus GI/GII | Excluded | Impacted by interventions | GI ORF1-2 and GII ORF1-2 |
| Sapovirus | Excluded | Impacted by interventions | *RdRp* |
| Adenovirus 40/41 | Excluded | Impacted by interventions | Fiber gene |
| **Environmental Enteric Dysfunction Biomarkers** | | |  |
| Fecal alpha-1-antitrypsin (age 14 months) | Included | NA | NA |
| Fecal myeloperoxidase (age 14 months) | Included | NA | NA |
| Fecal REG1B (age 14 months) | Included | NA | NA |
| Fecal lactulose and mannitol (age 3 months) | Excluded | Missingness greater than 30% | NA |
| Fecal myeloperoxidase (age 3 months) | Excluded | Missingness greater than 30% | NA |
| Fecal alpha-1-antitrypsin (age 3 months) | Excluded | Missingness greater than 30%, | NA |
| **Other Covariates** | | |  |
| Child sex | Included | NA | NA |
| Birth order | Included | NA | NA |
| Number of children under 18 years of age in the household | Included | NA | NA |
| Number of individuals in the compound (group of nearby houses) | Included | NA | NA |
| Household wall material | Included | NA | NA |
| Household wealth (first principal component of a principal components analysis incorporating household assets) | Included | NA | NA |
| Maternal age | Included | NA | NA |
| Maternal height | Included | NA | NA |
| Age (days) at urine and stool assessment | Included | NA | NA |
| Month of urine and stool assessment | Included | NA | NA |
| Age (days) at anthropometry assessment | Included | NA | NA |
| Age and month of assessment for stool and urine tests (at median age 3 months) | Excluded | Missingness greater than 30%, | NA |
| Maternal education | Excluded | Near zero variance | NA |
| Household food security | Excluded | Near zero variance | NA |
| Distance to water source | Excluded | Near zero variance | NA |
| Household floor material | Excluded | Near zero variance | NA |
