# Supplementary material for: Treatment heterogeneity of water, sanitation, hygiene, and nutrition interventions on child growth by environmental enteric dysfunction and pathogen status for young children in Bangladesh
Source: PLoS Negl Trop Dis. 2025 Feb 18;19(2):e0012881. doi: 10.1371/journal.pntd.0012881 (PMC11882089; doi:10.1371/journal.pntd.0012881)
Supplement: S1 Checklist — (DOCX) [file pntd.0012881.s015.docx]

**CONSORT Checklist**

| **Item** | **Description** | **Reported in Section** |
| --- | --- | --- |
| **Title and Abstract** | | |
| 1a | Identification as a randomized trial in the title; Identification as a cluster randomized trial in the title | N/A (this analysis used data from a substudy of the trial) |
| 1b | Structured summary of trial design, methods, results, and conclusions | Abstract |
| **Introduction** | | |
| Background and Objectives | | |
| 2a | Scientific background and explanation of rationale; Rationale for using a cluster design | Introduction |
| 2b | Specific objectives or hypotheses; Whether objectives pertain to the cluster level, the individual participant level, or both | Introduction |
| **Methods** | | |
| Trial Design | | |
| 3a | Description of trial design (such as parallel, factorial) including allocation ratio; Definition of cluster and description of how the design features apply to the clusters | Methods |
| 3b | Important changes to methods after trial commencement (such as eligibility criteria), with reasons | N/A |
| Participants | | |
| 4a | Eligibility criteria for participants; Eligibility criteria for clusters | Methods; Supplementary Materials |
| 4b | Settings and locations where the data were collected | Methods |
| Interventions | | |
| 5 | The interventions for each group with sufficient details to allow replication, including how and when they were actually administered; Whether interventions pertain to the cluster level, the individual participant level, or both | Methods; Supplementary Materials |
| Outcomes | | |
| 6a | Completely defined pre-specified primary and secondary outcome measures, including how and when they were assessed; Whether outcome measures pertain to the cluster level, the individual participant level, or both | N/A (secondary analysis of substudy of the trial) |
| 6b | Any changes to trial outcomes after the trial commenced, with reasons | N/A |
| Sample Size | | |
| 7a | How sample size was determined; Method of calculation, number of cluster(s) (and whether equal or unequal cluster sizes are assumed), cluster size, a coefficient of intracluster correlation (ICC or k), and an indication of its uncertainty | N/A (secondary analysis of substudy of the trial) |
| 7b | When applicable, explanation of any interim analyses and stopping guidelines | N/A |
| **Randomization** | | |
| Sequence Generation | | |
| 8a | Method used to generate the random allocation sequence | Methods |
| 8b | Type of randomization; details of any restriction (such as blocking and block size); Details of stratification or matching if used | Methods |
| Allocation Concealment Mechanism | | |
| 9 | Mechanism used to implement the random allocation sequence (such as sequentially numbered containers), describing any steps taken to conceal the sequence until interventions were assigned; Specification that allocation was based on clusters rather than individuals and whether allocation concealment (if any) was at the cluster level, the individual participant level, or both | Methods |
| Implementation | | |
| 10a | Who generated the random allocation sequence, who enrolled clusters, and who assigned clusters to interventions | Methods |
| 10b | Mechanism by which individual participants were included in clusters for the purposes of the trial (such as complete enumeration, random sampling) | Methods |
| 10c | From whom consent was sought (representatives of the cluster, or individual cluster members, or both) and whether consent was sought before or after randomization | Methods |
| Blinding | | |
| 11a | If done, who was blinded after assignment to interventions (for example, participants, care providers, those assessing outcomes)  and how | Methods |
| 11b | If relevant, description of the similarity of interventions | N/A |
| Statistical Methods | | |
| 12a | Statistical methods used to compare groups for primary and secondary outcomes; How clustering was taken into account | Methods; Supplementary Materials |
| 12b | Methods for additional analyses, such as subgroup analyses and adjusted analyses | Methods; Supplementary Materials |
| **Results** | | |
| Participant Flow | | |
| 13a | For each group, the numbers of participants/clusters who were randomly assigned, received intended treatment, and were analyzed for the primary outcome | Results; Supplementary Materials |
| 13b | For each group, losses and exclusions after randomization, together with reasons, for both clusters and individual cluster members | Results; Supplementary Materials |
| Recruitment | | |
| 14a | Dates defining the periods of recruitment and follow-up | Results |
| 14b | Why the trial ended or was stopped | N/A |
| Baseline Data | | |
| 15 | A table showing baseline demographic and clinical characteristics for each group; Baseline characteristics for the individual and cluster levels as applicable for each group | N/A (secondary analysis of substudy of the trial) |
| Numbers Analyzed | | |
| 16 | For each group, number of participants/clusters (denominator) included in each analysis and whether the analysis was by the original assigned groups | Methods; Tables; Supplementary Materials |
| Outcomes and Estimation | | |
| 17a | For each primary and secondary outcome, results for each group, and the estimated effect size and its precision (such as 95% confidence interval); Results at the individual and cluster levels as applicable and a coefficient of intracluster correlation (ICC or k) for each primary outcome | Results; Tables; Supplementary Materials |
| 17b | For binary outcome, presentation of both absolute and relative effect sizes is recommended | N/A |
| Ancillary Analyses | | |
| 18 | Results of any other analyses performed, including subgroup analyses and adjusted analyses, distinguishing pre-specified from exploratory | Results; Supplementary Materials |
| Harms | | |
| 19 | All important harms or unintended effects in each group | N/A |
| **Discussion** | | |
| Limitations | | |
| 20 | Trial limitations, addressing sources of potential bias, imprecision and, if relevant, multiplicity of analyses | Discussion |
| Generalizability | | |
| 21 | Generalizability (external validity, applicability) of the trial findings; Generalizability to clusters and/or individual participants (as relevant) | Discussion |
| Interpretation | | |
| 22 | Interpretation consistent with results, balancing benefits and harms, and considering other relevant evidence | Discussion |
| **Other Information** | | |
| Registration | | |
| 23 | Registration number and name of trial registry | Methods (ClinicalTrials.gov (NCT01590095)) |
| Protocol | | |
| 24 | Where the full trial protocol can be accessed, if available | N/A |
| Funding | | |
| 25 | Sources of funding and other support (such as supply of drugs), role of funders | Funding |
