# Supplementary material for: Treatment heterogeneity of water, sanitation, hygiene, and nutrition interventions on child growth by environmental enteric dysfunction and pathogen status for young children in Bangladesh
Source: PLoS Negl Trop Dis. 2025 Feb 18;19(2):e0012881. doi: 10.1371/journal.pntd.0012881 (PMC11882089; doi:10.1371/journal.pntd.0012881)
Supplement: Attachment — Submitted filename: PLOS NTD WASH BENEFITS Bangladesh subgroup analysis on HAZ.pdf [file pntd.0012881.s016.pdf]

Thank you for the opportunity to review this interesting manuscript presenting a subgroup analysis to identify children who many have benefited from interventions in the Bangladesh WASH Benefits trial. The authors present an interesting and very worthwhile attempt to more precisely identify populations who would experience linear growth benefits from the WSH and/or nutrition (N) interventions that were part of the trial.

Overall, the paper is very informative and will represent a useful addition to the literature. Below are comments regarding the writing and analysis that I hope will be helpful.

Major comments

OVERALL

1. The biomarkers:

The fecal biomarkers should be preceded by the word “fecal”. E.g., starting on p 5 instead of neopterin “fecal neopterin”, etc. Some biomarkers can be measured from the circulation.

Similarly please use the terms “urinary lactulose and mannitol” rather than lactulose and mannitol. To assist the reader who may not be familiar with the implications of urinary lactulose, In the introduction (p 5) introduce this as dual sugar testing post-ingestion of sugars (as these are not naturally produced molecules (as opposed to NEO and MPO for example)) to assess gut permeability. Please restrict discussion of lactulose and mannitol results to lactulose:mannitol ratio or % lactulose excretion as measures of permeability. Mannitol is not thought to be actively absorbed (see PMID: 29597877).

2. Please be careful about use of the word “growth” which implies growth over time. Please use terms such as “anthropometric assessments” to describe assessments at single time points. For example, Table 5 – use of “HAZ” in place of “growth” in headers

INTRODUCTION

3. The Introduction is lengthy and rambling and repeats information presented in the Methods section. The Discussion section is also long and rambling. Both could benefit from editing. For ex, the 3rd and 4th paragraphs and the 1st sentence of the 5th paragraph of the Introduction are somewhat redundant and can be merged and the 2nd paragraph on p 13 is repetitive to what was presented elsewhere.
4. P 3 2nd para last sentence – recommend striking this sentence. Afterall, it directly contradicts the next paragraph and underplays WASH Benefits and SHINE trials.

METHODS

5. The analysis focuses on the differing effect of the interventions (implemented in utero to sometime during the 2nd year of life (unclear from the methods on p 19 if it was to 1.5 years of age, 2 years of age, 2.5 years of age – would be good to clarify in the Supplemental Material 1))

by high vs low EED biomarker status and detected vs undetected enteric pathogen at 14 months of age (as a proxy of "chronic exposure to these factors throughout childhood") on "LAZ" at 28 months of age.

a. P 21 Outcomes section needs clarification – if the static height-for-age anthropometric measurement was used as the outcome please edit the text as such. Refrain from referring to HAZ as "growth" as that implies a measure over time such as delta HAZ. At 28 months of age the measure should have been HAZ, not LAZ. Perhaps the use of "LAZ" was just an oversight?
b. Please explain why delta HAZ was not used as that seems to be a preferable outcome for this analysis. Consider analysis including delta HAZ as an outcome.
c. The section "Analyzed biomarkers" on p 21 is redundant – suggest deleting. More importantly, here and elsewhere throughout the manuscript please refrain from referring to the pathogens as "biomarkers" (eg p 12 first paragraph of Discussion)
d. When I first read the Pathogens section on pp 20-21 I had the impression that a continuous quantification of the pathogens data was used in analysis based on the text in the 1st paragraph on p 21. But the beginning of the results indicated that a detection/non detection approach was used which makes sense given the analytic approach. It would be helpful to briefly state in the Methods that the pathogens data was dichotomized to detected/non-detected.
e. Did the authors consider examining the biomarkers data to compare the upper tercile or quartile to the lower tercile or quartile, respectively, in addition to the above and below the median approach? This could sharpen the ability to further identify differences in effect.
f. A high proportion of the children at the age when the fecal biomarkers and enteropathogens data was measured (14 months) had diarrhea per Table 1. Diarrhea status could be highly associated with the biomarkers (both because of gut pathophysiology of symptomatic diarrhea and dilution of the markers due to loose or watery stools) and enteropathogens. Recommend a sensitivity analysis excluding the children with diarrhea.

6. Pathogens analyzed
   a. There appear to be errors in Supplemental Material 5 which might be easily identified fi the authors prepare a table. For ex, the text includes "any enterotoxigenic *Escherichia coli* (ETEC; pathotypes *LT, STh, STp*), atypical enteropathogenic *Escherichia coli* (aEPEC; pathotypes (*eae* without *bfpA, stx1,* and *stx2*), typical enteropathogenic *Escherichia coli* (EPEC; pathotypes *bfpA* and *eae),… E. coli* with heat-stable toxin, Shiga toxin–producing *E. coli*". Presumably the authors meant "any enterotoxigenic *Escherichia coli* (ETEC; LT, STh, or STp), atypical enteropathogenic *E. coli* (aEPEC; *eae* without *bfpA*), typical enteropathogenic *E. coli* (EPEC; *bfpA* and *eae)*, … Shiga toxin-producing *E. coli* (stx1 or stx2)*"*. (Also term "pathotype seems to have been incorrectly used to refer to toxins, proteins, or genes in this example.)
   b. The text on p 21 states that the full list of pathogens are listed in Supplemental Material 5 and that 3 pathogens were excluded due to being identified as potentially mediators in a former analysis and 25 were excluded due to high missingness or

near-zero variance. These 28 + the 6 pathogens that were used in the current analysis are < the number listed in Supplemental Material 5. It would be good to convert the text in Supplemental Material 5 to a table and include a column indicating which were included and why those that were excluded were excluded.

   c. Supplemental Material 5 states "We assessed the relative concentration of the following pathogens". This can lead the reader to assume a continuous quantification of the pathogen was used as a variable rather than detected/not detected based on a cT cutpoint. Please clarify this sentence.

RESULTS

7. Unclear why "median age 28 months" for the outcome is stated. From the methods it sounded as though the HAZ at 28 months of age for all children was utilized. If not, please report age in months at Year 2 HAZ measure with IQR.
8. Recommend including a table (would become a new Table 2) with the biomarker and pathogen results at 14 months of age. This should include % missing, median, IQR, and range for the biomarkers and % missing and % positive for the enteropathogens. Also need to present how many of the children included in the analysis had none of the 6 enteropathogens analyzed, how many had only 1, 2, 3, etc. In a supplemental table would be helpful to also include co-infections with enteropathogens outside of the 6 that were utilized in this analysis.
   a. If high proportion with co-infections, please discuss implications in limitations section.
9. Avoid describing "high" enteropathogen vs "low" enteropathogen -- eg "comparing the treatment among children with high ETEC to children with low ETEC" on p 9. With and without ETEC or detected/non-detected would be more appropriate given the analytic approach by cT <35. It's ok to describe the biomarkers that way though.
10. Table 2-4 – it would be helpful to include a footnote explaining what the interpretation of the treatment effect #s mean and use an example of the first row (ETEC) to describe. Also including a column with N – the number of children including in each analysis row.
11. Table 5 – again footnotes with the interpretation of the data presented in the columns would be helpful. It's not very clear even when reading alongside the text.

DISCUSSION

12. P 12 1st paragraph 2nd sentence – burden does not seem like an appropriate term
13. P 12 last 2 sentences seem to have been copied and pasted from the Amour et al article and could benefit from some revision. Amour et al was suggesting targeted antibiotics as an intervention in their discussion but their results included macrolide use, breastfeeding, and access to water and san were associated with decreased risk of *Campylobacter* infection. The last sentence deserves actually describing the markers in their results because they very much overlap with those analyzed in WASH Benefits (including this analysis) rather than the processes they reflect that was used in the Amour et al abstract.
14. Would be good to weave in your thoughts on neopterin and L:M from your previous analyses into the discussion.
15. Can the authors speculate why WSH treatment only would have a bigger treatment difference compared to WSH + N (Campy and MPO)?

Minor comments:

GENERAL

1. Please check for appropriate utilization – for ex
    a. Abstract – Campylobacter should be italicized.
    b. Supplemental Material 5 "pathotypes *aaiC, or aatA, or bot" should be "*pathotypes *aaiC, aatA,* or both*"*
    c. Toxins such as LT, STh, STp do not need to be capitalized.
    d. Tables 2 -4 pathogen names need to be italicized.

INTRODUCTION

2. P 3 2nd para 1st sentence suggests malabsorption is the only mechanism by which pathogens lead to malnutrition is overly simplified – for ex, chronic systemic inflammation is thought to play a major role. Furthermore citations 7 and 8 do not support the text in the sentence.
3. Introduce abbreviations first use and use throughout
4. Please use a consistent tense in describing the literature. For ex, on p 5 the past tense is used except for describing SBBO association with outcomes which is described in present tense.
5. P 5 last full paragraph, while the sentence is almost a direct copy-paste from citation 15 abstract, it isn't clear as stated without the context of the rest of the abstract. For ex, could reword to something like: "SHINE Trial WSH interventions did not have an effect on bacterial, viral or parasitic enteropathogen prevalence although parasite concentrations were decreased in the stools of children in the intervention arm [15]." Even better, suggest moving the concept to the next paragraph which describes pathogens while the paragraph it is currently in describes EED markers.
6. P 6 sentence "At Year 2 (age 31 months), investigators found that individual sanitation and hygiene interventions were associated with decreased Giardia infections and that drinking water and nutrition interventions were not associated with a change in Giardia infections" – the term "change" is not clear in this context. Do the authors mean change in prevalence between time points? It doesn't seem so, it seems that the authors are drawing a contrast between prevalence association with san and hygiene but not water and nutrition interventions. And in the following sentence do the authors mean hookworm "prevalence"? Since the paper is not only about prevalence but also quantity or concentration of pathogens, would be good to be clear about these statements.
7. Starting at the bottom of page 6 and into page 7 there is awkward use of "we" as personal plural when describing the public health community as opposed to the authors' work. Suggest, as an ex, instead of on p 7 "We can assess the variance of…" to state "The variance of…can be assessed"

METHODS

8. P18 – it is noted that the laboratory teams were masked. Were the data analysts masked?
9. Supplemental Material 1 – please clarify if SQLNS was daily
10. Most of the text about the promoters in the Methods section pp 18-19 could be moved to Supplemental Material 1.
11. Also, the text currently reads as if the intervention was primarily health education by the promoters to encourage behavior change but the intervention as described in Supplemental Material 1 is more intensive and involved provision of materials to facilitate use of WSH.
12. Include biomarker kit information in Supplemental materials. Also how were values above and below the standard curve handled?

RESULTS

13. Sentence beginning on bottom of page 8/top p 9 seems redundant. Consider deleting.

DISCUSSION

14. P 16 Future directions section 1st paragraph is a bit awkwardly worded. Seems the authors' data support interventions targeting Campy generally, not just reducing exposure. Also, interventions to reduce intestinal inflammation, rather than interventions to reduce markers of inflammation.
