# Supplementary material for: Treatment heterogeneity of water, sanitation, hygiene, and nutrition interventions on child growth by environmental enteric dysfunction and pathogen status for young children in Bangladesh
Source: PLoS Negl Trop Dis. 2025 Feb 18;19(2):e0012881. doi: 10.1371/journal.pntd.0012881 (PMC11882089; doi:10.1371/journal.pntd.0012881)
Supplement: Attachment — Submitted filename: re-review PNTD-D-24-00822.docx [file pntd.0012881.s018.docx]

Thank you for the opportunity to re-review this is very informative paper. The revision is well done and I appreciate the attention to the response to reviewers’ comments.

Remaining points that would benefit from being addressed:

# Enteropathogens

Enteropathogens are a major part of the analysis in this paper. As such Supplementary Table 14 is appreciated but it is incomplete.

Please include the gene targets on the TAC card for each of the rows to allow comparison to past and future publications.

Please split out high missingness v near zero variance in the exclusion column as these are quite different from each other.

Minor points Supplemental Table 14– Entamoeba is abbreviated as E before the full name appears in the table. “spp” is not uniformly included where needed (e.g., *Salmonella spp.*).

What cycle threshold cutpoint was used to define pathogen detection vs non detection? Please include this in the methods.

In the results please avoid referring to pathogen data with descriptors such as “high” as the variable is analyzed as detected or not detected at a timepoint so saying “children with high ETEC to children with low ETEC” isn’t accurately portraying the data. Would be more appropriate to say “Children with ETEC to children without ETEC)”

Minor point Introduction – paragraph “Effect Measure Modification by EED and Pathogens” suggest slight revision to the last sentences: “Despite this lack of evidence of interaction, enteropathogen and environmental enteric dysfunction (EED) biomarker data may provide additional information on which subgroups of children, defined by pathogen infection/carriage status or biomarker levels, are amenable or resistant to intervention”

Minor point introduction – suggest adding “in their stools” to this sentence: “Investigation of the relationships between N+WSH interventions and enteropathogens at Year 1

(age 14 months) in Bangladesh found that children who received WSH interventions had a lower

prevalence and quantity of some individual viruses in their stools (norovirus, sapovirus, and adenovirus 40/41) compared to children in the control group”

# Anthropometry outcomes

I appreciate that the authors may not want to conduct a new analysis examining delta HAZ as an outcome but I found the response to the question in the initial review “Thank you for this question. We did not use delta HAZ as an outcome due to concerns regarding regression to the mean and catchup growth [CITE]” a bit superficial and dismissive. There are good reasons to why delta HAZ is a better outcome measure than static HAZ. Please discuss the pros and cons in the limitations section of the discussion.

# Other results

The new sentence that was added in the Results under “Post-hoc analysis” – “To improve interpretability, we dichotomized *Campylobacter* based on detection (non-zero value) vs. non-detection (zero value), while we dichotomized myeloperoxidase as high concentration (above median value) vs. low concentration (below median value). “ is confusing as my read of the paper is that all of the pathogens were analyzed dichotomized (detected or non-detected). It seems that the new analytic point being raised here is the dichotomization of the biomarker. Please clarify the sentence – maybe as simple as “To improve interpretability, we dichotomized myeloperoxidase as high concentration (above median value) vs. low concentration (below median value) (and retained *Campylobacter* as dichotomized based on detection (non-zero value) vs. non-detection (zero value).” I’m not sure the “non-zero value” and “zero value” in parentheses adds helpful information? If the authors have good rationale for this parenthetic clarification, that is fine.

Thank you for adding Supplemental Table 7 with the sensitivity analysis excluding children with diarrhea.

The text describing this analysis deserves it’s own paragraph in the results section as currently it follows a paragraph on Campy infection & myeloperoxidase only and makes it seems that the diarrhea sensitivity analysis was only regarding this Campy/myeloperoxidase analysis.

Minor point – in the header in Supp Table 7, avoid use of word “diarrhea cases” as it implies that only the cases and not controls were considered in this sensitivity analysis. Consider “Excluding Children with Diarrhea” or something like that instead.

A comment in the original review was “Recommend including a table (would become a new Table 2) with the biomarker and pathogen results at 14 months of age. This should include % missing, median, IQR, and range for the biomarkers and % missing and % positive for the enteropathogens. Also need to present how many of the children included in the analysis had none of the 6 enteropathogens analyzed, how many had only 1, 2, 3, etc. In a supplemental table would be helpful to also include co-infections

with enteropathogens outside of the 6 that were utilized in this analysis.” And the response was

a. If high proportion with co-infections, please discuss implications in limitations section.

“b. Published elsewhere – cite previous study

c. Thank you for this question. A previous study evaluated the impact of the interventions on

child pathogens and child EED biomarkers, so we have added a more detailed description of

their findings and how they relate to our research question (pages 5-6).”

I do not see where this question was addressed or the citation even after looking through pages 5-6. Please directly copy – paste the text in the manuscript that addresses these questions (including if the text references pre-existing publication, which is fine).

# Discussion

The term “severity” that has been added in the 2^nd^ sentence of the 1^st^ paragraph is misleading. More severe Campy infection implies that a child with Campy has more severe signs and symptoms. Seems that this should be “with *Campylobacter* and higher myeloperoxidase”.
