# Supplementary material for: Treatment heterogeneity of water, sanitation, hygiene, and nutrition interventions on child growth by environmental enteric dysfunction and pathogen status for young children in Bangladesh
Source: PLoS Negl Trop Dis. 2025 Feb 18;19(2):e0012881. doi: 10.1371/journal.pntd.0012881 (PMC11882089; doi:10.1371/journal.pntd.0012881)
Supplement: Attachment — Submitted filename: OTR External Reviewer Responses.docx [file pntd.0012881.s019.docx]

Thank you for the opportunity to review this interesting manuscript presenting a subgroup analysis to identify children who many have beneﬁted from interventions in the Bangladesh WASH Beneﬁts trial. The authors present an interesting and very worthwhile attempt to more precisely identify populations who would experience linear growth beneﬁts from the WSH and/or nutrition (N) interventions that were part of the trial.

Overall, the paper is very informative and will represent a useful addition to the literature. Below are comments regarding the writing and analysis that I hope will be helpful.

Thank you so much for your careful review and feedback. We have incorporated your suggestions into our manuscript and materials, and we believe that your comments have greatly strengthened this study. We are grateful for your time and consideration.

Major comments OVERALL

1. The biomarkers:

The fecal biomarkers should be preceded by the word “fecal”. E.g., starting on p 5 instead of neopterin “fecal neopterin”, etc. Some biomarkers can be measured from the circulation.

Thank you for this comment. We have corrected our description of biomarkers as suggested.

Similarly please use the terms “urinary lactulose and mannitol” rather than lactulose and mannitol. To assist the reader who may not be familiar with the implications of urinary lactulose, In the introduction (p 5) introduce this as dual sugar testing post-ingestion of sugars (as these are not naturally produced molecules (as opposed to NEO and MPO for example)) to assess gut permeability. Please restrict discussion of lactulose and mannitol results to lactulose:mannitol ratio or % lactulose excretion as measures of permeability. Mannitol is not thought to be actively absorbed (see PMID: 29597877).

Thank you for sharing this context. We have corrected our description of “urinary lactulose and mannitol” and have limited our introduction to only describe this ratio.

2. Please be careful about use of the word “growth” which implies growth over time. Please use terms such as “anthropometric assessments” to describe assessments at single time points. For example, Table 5 – use of “HAZ” in place of “growth” in headers

Thank you for this note. I have edited the text and tables to replace “growth” with “HAZ” or “anthropometry” throughout.

## INTRODUCTION

3. The Introduction is lengthy and rambling and repeats information presented in the Methods section. The Discussion section is also long and rambling. Both could beneﬁt from editing. For ex, the 3^rd^ and 4^th^ paragraphs and the 1^st^ sentence of the 5^th^ paragraph of the Introduction are somewhat redundant and can be merged and the 2^nd^ paragraph on p 13 is repetitive to what was presented elsewhere.
   a. Thank you for these comments. We significantly reduced the introduction, methods, and discussion as suggested with the aim of improving flow and reducing redundancy.
4. P 3 2^nd^ para last sentence – recommend striking this sentence. Afterall, it directly contradicts the next paragraph and underplays WASH Beneﬁts and SHINE trials.
   a. Thank you for this suggestion. We removed this sentence as recommended.

## METHODS

5. The analysis focuses on the diﬀering eﬀect of the interventions (implemented in utero to sometime during the 2^nd^ year of life (unclear from the methods on p 19 if it was to 1.5 years of age, 2 years of age, 2.5 years of age – would be good to clarify in the Supplemental Material 1)
   a. Thank you for this comment. We added specification that interventions continued until child age 24 months (page 16, Supplement 1).

by high vs low EED biomarker status and detected vs undetected enteric pathogen at 14 months of age (as a proxy of “chronic exposure to these factors throughout childhood”) on “LAZ” at 28 months of age.

a. P 21 Outcomes section needs clariﬁcation – if the static height-for-age anthropometric measurement was used as the outcome please edit the text as such. Refrain from referring to HAZ as “growth” as that implies a measure over time such as delta HAZ. At 28 months of age the measure should have been HAZ, not LAZ. Perhaps the use of “LAZ” was just an oversight?
   - Thank you for this note and correction. We have corrected “LAZ” to “HAZ” throughout, given that our primary outcome was child anthropometry at 28 months.
b. Please explain why delta HAZ was not used as that seems to be a preferable outcome for this analysis. Consider analysis including delta HAZ as an outcome.
   - Thank you for this question. We did not use delta HAZ as an outcome due to concerns regarding regression to the mean and catchup growth [CITE]
c. The section “Analyzed biomarkers” on p 21 is redundant – suggest deleting. More importantly, here and elsewhere throughout the manuscript please refrain from referring to the pathogens as “biomarkers” (eg p 12 ﬁrst paragraph of Discussion)
   - Thank you for these suggestions. We removed the referenced paragraph and rephrased throughout to avoid referring to pathogens as biomarkers.
d. When I ﬁrst read the Pathogens section on pp 20-21 I had the impression that a continuous quantiﬁcation of the pathogens data was used in analysis based on the text in the 1^st^ paragraph on p 21. But the beginning of the results indicated that a detection/non detection approach was used which makes sense given the analytic approach. It would be helpful to brieﬂy state in the Methods that the pathogens data was dichotomized to detected/non-detected.
   - Thank you for this question. In our main analysis (developing an optimal treatment rule and determining child anthropometry under this rule), we used continuous values of pathogen and biomarker data. For interpretability, we reported the counterfactual difference in child anthropometry at each level of child anthropometry. We added details to the methods to make this distinction more clear.
e. Did the authors consider examining the biomarkers data to compare the upper tercile or quartile to the lower tercile or quartile, respectively, in addition to the above and below the median approach? This could sharpen the ability to further identify diﬀerences in eﬀect.
   - Thank you for this question. We considered using alternative quantile-based comparisons in our analysis. After reviewing the distributions of biomarker and pathogen values in our sample, and discussing these distributions with co-authors who are subject-matter experts, we determined that the above vs below the median approach would be the most informative for our research question.
f. A high proportion of the children at the age when the fecal biomarkers and enteropathogens data was measured (14 months) had diarrhea per Table 1. Diarrhea status could be highly associated with the biomarkers (both because of gut pathophysiology of symptomatic diarrhea and dilution of the markers due to loose or watery stools) and enteropathogens. Recommend a sensitivity analysis excluding the children with diarrhea.
   - Thank you for this suggestion. We conducted a sensitivity analysis as suggested, and found that excluding children with diarrhea did not impact our results or inference (page 9, supplemental material X)

7. Pathogens analyzed
   a. There appear to be errors in Supplemental Material 5 which might be easily identiﬁed ﬁ the authors prepare a table. For ex, the text includes “any enterotoxigenic *Escherichia coli* (ETEC; pathotypes *LT, STh, STp*), atypical enteropathogenic *Escherichia coli* (aEPEC; pathotypes (*eae* without *bfpA, stx1,* and *stx2*), typical enteropathogenic *Escherichia coli* (EPEC; pathotypes *bfpA* and *eae),… E. coli* with heat-stable toxin, Shiga toxin–producing *E. coli*”. Presumably the authors meant “any enterotoxigenic *Escherichia coli* (ETEC; LT, STh, or STp), atypical enteropathogenic *E. coli* (aEPEC; *eae* without *bfpA*), typical enteropathogenic *E. coli* (EPEC; *bfpA* and *eae)*, … Shiga toxin- producing *E. coli* (stx1 or stx2)*”*. (Also term “pathotype seems to have been incorrectly used to refer to toxins, proteins, or genes in this example.)
      - Thank you for highlighting these errors. We added a new supplemental table, as suggested, to track pathogen exclusion, and corrected terminology as indicated.

# b. The text on p 21 states that the full list of pathogens are listed in Supplemental Material 5 and that 3 pathogens were excluded due to being identiﬁed as potentially mediators in a former analysis and 25 were excluded due to high missingness or

## RESULTS

near-zero variance. These 28 + the 6 pathogens that were used in the current analysis are < the number listed in Supplemental Material 5. It would be good to convert the text in Supplemental Material 5 to a table and include a column indicating which were included and why those that were excluded were excluded.

Thank you for this comment. We converted Supplemental Material 5 to a table, as suggested, indicating which pathogens were excluded.

# c. Supplemental Material 5 states “We assessed the relative concentration of the following pathogens”. This can lead the reader to assume a continuous quantiﬁcation of the pathogen was used as a variable rather than detected/not detected based on a cT cutpoint. Please clarify this sentence.

Thank you for this suggestion. We corrected this sentence as requested.

8. Unclear why “median age 28 months” for the outcome is stated. From the methods it sounded as though the HAZ at 28 months of age for all children was utilized. If not, please report age in months at Year 2 HAZ measure with IQR.
   a. Thank you for this question. As this was a large, randomized trial, the age at data collection was not identical for all participants. We added the age distribution at Year 2 anthropometric assessment: median age 28.1 months (IQR 26.8 months, 29.2 months) to page 6.
9. Recommend including a table (would become a new Table 2) with the biomarker and pathogen results at 14 months of age. This should include % missing, median, IQR, and range for the biomarkers and % missing and % positive for the enteropathogens. Also need to present how many of the children included in the analysis had none of the 6 enteropathogens analyzed, how many had only 1, 2, 3, etc. In a supplemental table would be helpful to also include co-infections with enteropathogens outside of the 6 that were utilized in this analysis.
   a. If high proportion with co-infections, please discuss implications in limitations section.
   b. Published elsewhere – cite previous study
   c. Thank you for this question. A previous study evaluated the impact of the interventions on child pathogens and child EED biomarkers, so we have added a more detailed description of their findings and how they relate to our research question (pages 5-6).
10. Avoid describing “high” enteropathogen vs “low” enteropathogen -- eg “comparing the treatment among children with high ETEC to children with low ETEC” on p 9. With and without ETEC or detected/non-detected would be more appropriate given the analytic approach by cT

<35. It’s ok to describe the biomarkers that way though.

Thank you for this suggestion. We have corrected our description of pathogens as suggested.

11. Table 2-4 – it would be helpful to include a footnote explaining what the interpretation of the treatment eﬀect #s mean and use an example of the ﬁrst row (ETEC) to describe. Also including a column with N – the number of children including in each analysis row.
    a. Thank you for this comment. We added an example interpretation below each table as suggested.
12. Table 5 – again footnotes with the interpretation of the data presented in the columns would be helpful. It’s not very clear even when reading alongside the text.
    a. Thank you for this comment. We added an example interpretation below each table as suggested.

## DISCUSSION

13. P 12 1^st^ paragraph 2^nd^ sentence – burden does not seem like an appropriate term
    a. Thank you for this suggestion. We have replaced “burden” with “severity” here.
14. P 12 last 2 sentences seem to have been copied and pasted from the Amour et al article and could beneﬁt from some revision. Amour et al was suggesting targeted antibiotics as an intervention in their discussion but their results included macrolide use, breastfeeding, and access to water and san were associated with decreased risk of *Campylobacter* infection. The last sentence deserves actually describing the markers in their results because they very much overlap with those analyzed in WASH Beneﬁts (including this analysis) rather than the processes they reﬂect that was used in the Amour et al abstract.
    a. Thank you for highlighting this excerpt. We added more specific details from this article to describe the authors’ findings in terms of biomarkers.
15. Would be good to weave in your thoughts on neopterin and L:M from your previous analyses into the discussion.
    a. Thank you for this suggestion. We have added additional discussion of the biological implications of our findings. As we excluded neopterin and L:M from our analyses, we do not believe that it is necessary to include a discussion of these factors in the discussion of this paper.
16. Can the authors speculate why WSH treatment only would have a bigger treatment diﬀerence compared to WSH + N (Campy and MPO)?

Thank you for this question. The greater magnitude of the treatment effect difference given *Campylobacter* detection and above median myeloperoxidase versus *Campylobacter* non-detection and below median myeloperoxidase for WSH, compared to N+WSH, may be attributable to finite sample bias, in which the observed LAZ in the WSH arm was lower than the other three treatment arms. This large treatment effect difference for WSH vs. control may be attributable to the wide variation in anthropometry observed in the WSH arm, which makes this group amenable to an optimized intervention. This subsample did not retain the same anthropometric characteristics as the main sample. We have added these considerations to our discussion on page 14.

Minor comments:

## GENERAL

1. Please check for appropriate utilization – for ex
   a. Abstract – Campylobacter should be italicized.
      - Thank you for this comment. We have updated accordingly.
   b. Supplemental Material 5 “pathotypes *aaiC, or aatA, or bot” should be “*pathotypes *aaiC, aatA,* or both*”*
      - Thank you for this suggestion. We have updated accordingly.
   c. Toxins such as LT, STh, STp do not need to be capitalized.
      - Thank you for this note. We have edited these excerpts.
   d. Tables 2 -4 pathogen names need to be italicized.
      - Thank you for this note. We have updated accordingly.

## INTRODUCTION

2. P 3 2^nd^ para 1^st^ sentence suggests malabsorption is the only mechanism by which pathogens lead to malnutrition is overly simpliﬁed – for ex, chronic systemic inﬂammation is thought to play a major role. Furthermore citations 7 and 8 do not support the text in the sentence.
   a. Thank you for this comment. We have updated this excerpt to reflect other potential pathways, and we have replaced these references with more relevant sources.
3. Introduce abbreviations ﬁrst use and use throughout
   a. Thank you for this note. We have edited the manuscript accordingly.
4. Please use a consistent tense in describing the literature. For ex, on p 5 the past tense is used except for describing SBBO association with outcomes which is described in present tense.
   a. Thank you for this note. We have corrected this excerpt and other similar errors.
5. P 5 last full paragraph, while the sentence is almost a direct copy-paste from citation 15 abstract, it isn’t clear as stated without the context of the rest of the abstract. For ex, could reword to something like: “SHINE Trial WSH interventions did not have an eﬀect on bacterial, viral or parasitic enteropathogen prevalence although parasite concentrations were decreased in the stools of children in the intervention arm [15].” Even better, suggest moving the concept to the next paragraph which describes pathogens while the paragraph it is currently in describes EED markers.
   a. Thank you for this suggestion. We have edited as recommended.
6. P 6 sentence “At Year 2 (age 31 months), investigators found that individual sanitation and hygiene interventions were associated with decreased Giardia infections and that drinking water and nutrition interventions were not associated with a change in Giardia infections” – the term “change” is not clear in this context. Do the authors mean change in prevalence between time points? It doesn’t seem so, it seems that the authors are drawing a contrast between prevalence association with san and hygiene but not water and nutrition interventions. And in the following sentence do the authors mean hookworm “prevalence”? Since the paper is not only about prevalence but also quantity or concentration of pathogens, would be good to be clear about these statements.
   a. Thank you for this question. Here, “change” refers to the contrast between treatment and control groups. I have added details here to describe these contrasts.
7. Starting at the bottom of page 6 and into page 7 there is awkward use of “we” as personal plural when describing the public health community as opposed to the authors’ work. Suggest, as an ex, instead of on p 7 “We can assess the variance of…” to state “The variance of…can be assessed”

Thank you for this comment. I moved this section to Supplemental Material and rephrased these portions as suggested.

## METHODS

8. P18 – it is noted that the laboratory teams were masked. Were the data analysts masked?
   a. Thank you for this question. It was not possible to mask data analysts for this study.
9. Supplemental Material 1 – please clarify if SQLNS was daily
   a. Thank you for this question. SQLNS was given daily, and we have updated Supplemental Material 1 accordingly.
10. Most of the text about the promoters in the Methods section pp 18-19 could be moved to Supplemental Material 1.
    a. Thank you for this suggestion. We moved these details about the promoters to Supplemental Material 1.
11. Also, the text currently reads as if the intervention was primarily health education by the promoters to encourage behavior change but the intervention as described in Supplemental Material 1 is more intensive and involved provision of materials to facilitate use of WSH.
    a. Thank you for this question. We have streamlined and added details on the interventions (page 16) with additional details in supplemental material 1.
12. Include biomarker kit information in Supplemental materials. Also how were values above and below the standard curve handled?
    a. Thank you for this question. We added this information to Supplemental Material 4.

## RESULTS

13. Sentence beginning on bottom of page 8/top p 9 seems redundant. Consider deleting.
    a. Thank you for noting this redundancy. We deleted as suggested.
14. DISCUSSION
15. P 16 Future directions section 1^st^ paragraph is a bit awkwardly worded. Seems the authors’ data support interventions targeting Campy generally, not just reducing exposure. Also, interventions to reduce intestinal inﬂammation, rather than interventions to reduce markers of inﬂammation.
    a. Thank you for this note. We edited this excerpt to improve clarity.
