# Supplementary material for: Treatment heterogeneity of water, sanitation, hygiene, and nutrition interventions on child growth by environmental enteric dysfunction and pathogen status for young children in Bangladesh
Source: PLoS Negl Trop Dis. 2025 Feb 18;19(2):e0012881. doi: 10.1371/journal.pntd.0012881 (PMC11882089; doi:10.1371/journal.pntd.0012881)
Supplement: Attachment — Submitted filename: OTR Reviewer Responses_1.22.25.docx [file pntd.0012881.s020.docx]

*Dear Reviewers,*

*We greatly appreciate your careful review and helpful feedback. We have included the reviewers’ comments below (normal text, indented), where we have marked our responses using italics (left justified).*

Thank you for the opportunity to re-review this is very informative paper. The revision is well done and I appreciate the attention to the response to reviewers’ comments.

Remaining points that would benefit from being addressed:

In addition to the comments attached to this decision, the reviewer also noted that if a ct cut-off other than <35 was used, they believe it would be appropriate to conduct a sensitivity analysis using that cutoff. Please also consider this comment in your revisions.

*Thank you for this comment. We did use the cut-off of <35, and we added this detail to Supplemental Material 13. We rephrased the following text on page 20 for clarity: “We quantified pathogens using quantification cycle, where one unit corresponded to twice the pathogen quantity. The analytical limit of detection was quantification cycle 35, values above this were considered non-detects [64].”*

# Enteropathogens

Enteropathogens are a major part of the analysis in this paper. As such Supplementary Table 14 is appreciated but it is incomplete.

Please include the gene targets on the TAC card for each of the rows to allow comparison to past and future publications.

*Thank you for this suggestion. We added a column titled “Gene target” to Supplemental Material 14 that includes this information.*

Please split out high missingness v near zero variance in the exclusion column as these are quite different from each other.

*Thank you for this question, and we apologize for this vague phrasing. We have updated Supplemental Material 14 with more specific exclusion reasons.*

Minor points Supplemental Table 14– Entamoeba is abbreviated as E before the full name appears in the table. “spp” is not uniformly included where needed (e.g., *Salmonella spp.*).

*Thank you for this comment. We corrected Supplemental Table 14 to spell out the full genus and to add “spp” where necessary.*

What cycle threshold cutpoint was used to define pathogen detection vs non detection? Please include this in the methods.

*Thank you for this question. We quantified pathogens using quantification cycle, where one unit corresponded to twice the pathogen quantity and there was an analytical limit of detection at quantification cycle 35 (Supplemental material 13). We rephrased the following text on page 20 for clarity: “We quantified pathogens using quantification cycle, where one unit corresponded to twice the pathogen quantity. The analytical limit of detection was quantification cycle 35, values above this were considered non-detects [64].”.*

In the results please avoid referring to pathogen data with descriptors such as “high” as the variable is analyzed as detected or not detected at a timepoint so saying “children with high ETEC to children with low ETEC” isn’t accurately portraying the data. Would be more appropriate to say “Children with ETEC to children without ETEC)”

*Thank you for this suggestion. We updated this excerpt accordingly (page 8).*

Minor point Introduction – paragraph “Effect Measure Modification by EED and Pathogens” suggest slight revision to the last sentences: “Despite this lack of evidence of interaction, enteropathogen and environmental enteric dysfunction (EED) biomarker data may provide additional information on which subgroups of children, defined by pathogen infection/carriage status or biomarker levels, are amenable or resistant to intervention”

*Thank you for this comment. We updated this excerpt according to your suggestion.*

Minor point introduction – suggest adding “in their stools” to this sentence: “Investigation of the relationships between N+WSH interventions and enteropathogens at Year 1 (age 14 months) in Bangladesh found that children who received WSH interventions had a lower prevalence and quantity of some individual viruses in their stools (norovirus, sapovirus, and adenovirus 40/41) compared to children in the control group”

*Thank you for noting this detail. We added “in their stools” to this sentence as suggested.*

# Anthropometry outcomes

I appreciate that the authors may not want to conduct a new analysis examining delta HAZ as an outcome but I found the response to the question in the initial review “Thank you for this question. We did not use delta HAZ as an outcome due to concerns regarding regression to the mean and catchup growth [CITE]” a bit superficial and dismissive. There are good reasons to why delta HAZ is a better outcome measure than static HAZ. Please discuss the pros and cons in the limitations section of the discussion.

*Thank you for your comment, and we apologize for our insufficient incorporation of your feedback. We added an excerpt to the limitations section regarding our use of HAZ. We have pasted this paragraph below, with the new text highlighted in bold.*

*“One limitation of this study arises from using post-intervention biomarkers and pathogens, as no baseline EED biomarkers or pathogens were measured because infants were in utero at the time of randomization. Conditioning on these post-intervention nodes potentially introduces confounding and bias. We accounted for this possible confounding by adjusting for additional baseline covariate information related to family health and socioeconomic status and by excluding pathogens and EED biomarkers that were associated with the interventions in previous analyses of this sample (i.e., potential mediators or colliders), although residual confounding or bias may be present.* ***Using growth velocity evaluated after biomarker assessment, rather than HAZ, as an outcome of interest may alleviate these concerns in some settings, but it would have considerable drawbacks in our study context. As our interventions were delivered at birth, we do not have pre-intervention anthropometric assessments, which would be required to assess the full impact of the interventions on growth velocity. Furthermore, as we randomized in-utero, we can infer that anthropometric characteristics were balanced at baseline. While it would be feasible to calculate growth velocity as the change in growth between Year 1 and Year 2, this presents additional inferential challenges, as children with early life growth faltering may experience subsequent “catchup growth” due to regression to the mean (convergence towards average trajectory) and/or due to post-birth biological recovery.[55,56] Therefore, given the effective randomization of this study, we selected HAZ as our outcome of interest.****”*

# Other results

The new sentence that was added in the Results under “Post-hoc analysis” – “To improve interpretability, we dichotomized *Campylobacter* based on detection (non-zero value) vs. non-detection (zero value), while we dichotomized myeloperoxidase as high concentration (above median value) vs. low concentration (below median value). “ is confusing as my read of the paper is that all of the pathogens were analyzed dichotomized (detected or non-detected). It seems that the new analytic point being raised here is the dichotomization of the biomarker. Please clarify the sentence – maybe as simple as “To improve interpretability, we dichotomized myeloperoxidase as high concentration (above median value) vs. low concentration (below median value) (and retained *Campylobacter* as dichotomized based on detection (non-zero value) vs. non-detection (zero value).” I’m not sure the “non-zero value” and “zero value” in parentheses adds helpful information? If the authors have good rationale for this parenthetic clarification, that is fine.

*Thank you for this comment. Under the optimal treatment rule, we incorporated continuous values for both EED biomarkers and pathogens in order to assess potential nonlinear or non-monotonic relationships. To improve interpretability in our assessment of variable importance, we summarized these factors dichotomously.*

Thank you for adding Supplemental Table 7 with the sensitivity analysis excluding children with diarrhea.

The text describing this analysis deserves it’s own paragraph in the results section as currently it follows a paragraph on Campy infection & myeloperoxidase only and makes it seems that the diarrhea sensitivity analysis was only regarding this Campy/myeloperoxidase analysis.

*Thank you for this suggestion. We moved this excerpt to its own paragraph to improve clarity.*

Minor point – in the header in Supp Table 7, avoid use of word “diarrhea cases” as it implies that only the cases and not controls were considered in this sensitivity analysis. Consider “Excluding Children with Diarrhea” or something like that instead.

*Thank you for this comment. We edited Supplemental Table 7 according to your suggestion.*

A comment in the original review was “Recommend including a table (would become a new Table 2) with the biomarker and pathogen results at 14 months of age. This should include % missing, median, IQR, and range for the biomarkers and % missing and % positive for the enteropathogens. Also need to present how many of the children included in the analysis had none of the 6 enteropathogens analyzed, how many had only 1, 2, 3, etc. In a supplemental table would be helpful to also include co-infections

with enteropathogens outside of the 6 that were utilized in this analysis.” And the response was

a. If high proportion with co-infections, please discuss implications in limitations section.

“b. Published elsewhere – cite previous study

c. Thank you for this question. A previous study evaluated the impact of the interventions on

child pathogens and child EED biomarkers, so we have added a more detailed description of

their findings and how they relate to our research question (pages 5-6).”

I do not see where this question was addressed or the citation even after looking through pages 5-6. Please directly copy – paste the text in the manuscript that addresses these questions (including if the text references pre-existing publication, which is fine).

*Thank you for this question. We have pasted the text from our manuscript below that describes these characteristics*

*“Previous analyses of the WASH Benefits Bangladesh study evaluated the impact of interventions on child pathogens and EED biomarkers. The investigators found that the nutrition intervention was associated with reduced fecal neopterin at 3 and 14 months of age, and all interventions reduced urinary lactulose and mannitol at 3 and 14 months [24]. The urinary lactulose to mannitol ratio quantifies the dual sugar absorption test to measure gut permeability.[25] At 28 months, contrary to a-priori hypotheses, WSH and nutrition interventions were associated with increased fecal myeloperoxidase [24]. Although these findings at ages 3 and 14 months support N+WSH interventions’ ability to reduce some EED biomarkers, the counterintuitive results at 28 months highlight uncertainty regarding the relationship between N+WSH interventions and presumed biomarkers for EED.*

*Investigators of the WASH Benefits study suggested that insufficient reduction of pathogen exposure could explain the null effects of WSH interventions on child linear growth [10]. Investigation of the relationships between N+WSH interventions and enteropathogens at Year 1 (age 14 months) in Bangladesh found that children who received WSH interventions had a lower prevalence and quantity of some individual viruses in their stools (norovirus, sapovirus, and adenovirus 40/41) compared to children in the control group, although investigators did not find a significant difference in bacteria, parasites, or stunting-related pathogens between these groups [13]. Furthermore, this study found that 99% of children at Year 1 had at least one enteropathogen [13].”*

*You can find a link to the previously published table (table 3, page 744) of EED results at 14 months here: https://www.researchgate.net/publication/332409194_Effects_of_Water_Sanitation_Handwashing_and_Nutritional_Interventions_on_Environmental_Enteric_Dysfunction_in_Young_Children_A_Cluster-randomized_Controlled_Trial_in_Rural_Bangladesh/fulltext/63e63604dea61217579bbe65/Effects-of-Water-Sanitation-Handwashing-and-Nutritional-Interventions-on-Environmental-Enteric-Dysfunction-in-Young-Children-A-Cluster-randomized-Controlled-Trial-in-Rural-Bangladesh.pdf*

*You can find a link to the previously published table (table 3) of pathogen results at 14 months here:* [*https://academic.oup.com/view-large/401476844*](https://academic.oup.com/view-large/401476844) *[13].*

Discussion

The term “severity” that has been added in the 2^nd^ sentence of the 1^st^ paragraph is misleading. More severe Campy infection implies that a child with Campy has more severe signs and symptoms. Seems that this should be “with *Campylobacter* and higher myeloperoxidase”.

*Thank you for this note. We have edited this excerpt according to your suggestion.*
